# Knowledge and Attitudes of Student Pharmacists Regarding Polypharmacy and Deprescribing: A Cross-Sectional Study

**DOI:** 10.3390/pharmacy8040220

**Published:** 2020-11-18

**Authors:** Collin M. Clark, Mary Hejna, Elaine Shao, Jaime L. Maerten-Rivera, Scott V. Monte, Robert G. Wahler

**Affiliations:** Department of Pharmacy Practice, School of Pharmacy and Pharmaceutical Sciences, University at Buffalo, Buffalo, NY 14214, USA; maryhejn@buffalo.edu (M.H.); eshao@buffalo.edu (E.S.); jmaerten@buffalo.edu (J.L.M.-R.); svmonte@buffalo.edu (S.V.M.); rgwahler@buffalo.edu (R.G.W.J.)

**Keywords:** deprescribing, polypharmacy, pharmacy, education, geriatrics, survey research

## Abstract

Pharmacists play a key role in deprescribing medications. Incorporation of this concept into pharmacy school curricula is important in ensuring that graduates can address the complex needs of an aging population. The aims of this study were to assess if and how student pharmacists were exposed to deprescribing within their curriculum, to assess students’ perceptions regarding their attitudes, ability and confidence in deprescribing, and to assess if reported curricular exposure to this topic resulted in improved perceptions or objective knowledge assessment scores. An electronic survey was distributed to third- and fourth-year pharmacy students at 132 schools of pharmacy. The survey included three sections including: (i) demographics and questions on their exposure to deprescribing and other experiences within their curriculum; (ii) questions regarding their attitudes, ability, and confidence regarding deprescribing on a 5-point Likert-scale; (iii) a knowledge assessment on polypharmacy and deprescribing in the form of 12 multiple-choice questions. Likert-scale questions were analyzed as scales utilizing the mean score for items measuring student perceptions regarding deprescribing attitudes, ability, and confidence. Comparisons were made on each variable between students with and without curricular exposure to deprescribing using *t*-tests. Ninety-one responses were included in the analysis. Only 59.3% of respondents reported exposure to deprescribing in their didactic coursework. The mean scores on the polypharmacy and deprescribing knowledge assessments were 61.0% and 64.5%, respectively. Those with exposure to deprescribing concepts within their curriculum were more likely to agree that their school’s curriculum prepared them to deprescribe in clinical practice (*t*(89) = −2.26, *p* = 0.03). Pharmacy schools should evaluate their curricula and consider the addition of specific deprescribing objectives and outcome measures for didactic and experiential training.

## 1. Introduction

Polypharmacy, defined as the use of multiple medications, is recognized as a risk factor for adverse drug reactions and healthcare utilization among older adults [1,2,3]. Polypharmacy is also associated with prescribing potentially inappropriate medications (PIMs), those in which the potential harms outweigh the benefits for most older adults [4,5]. As the percentage of the population over the age of 65 years old continues to grow, the need for healthcare professionals trained to take care of this population will likewise increase [6]. Deprescribing, defined as “a systematic process of identifying and discontinuing medications in instances in which existing potential harms outweigh existing or potential benefits within the context of an individual patient’s care goals, current level of functioning, life expectancy, values, and preferences,” is a term that is gaining traction throughout the medical community [7,8]. Deprescribing is a complex task that includes clinical knowledge, shared decision making with patients and caregivers, and communication between all care providers [8]. Pharmacists are uniquely positioned and trained to regularly assess a patient’s medication regimen and identify older adults at risk for adverse drug events [9]. In the United States (US), the entry level degree to practice pharmacy is the Doctor of Pharmacy (PharmD) and, in addition to medicine distribution activities, pharmacists routinely practice in clinical positions, in which they work with medical providers to optimize medication therapies. Several recent studies have demonstrated the value of pharmacists facilitating deprescribing across the continuum of care [10,11,12,13]. Challenges to deprescribing include lack of time and funding to complete such activities, patient expectations, and limited research and education related to deprescribing [14,15]. It has been suggested that a key change to increase the practice of deprescribing is educating health care providers at the undergraduate, graduate, and continuing professional education levels, which would include instruction on deprescribing as part of the curricula in medical and pharmacy education [16,17].

Previous studies over the last few decades have consistently shown that the extent to which geriatric content is taught at US schools of pharmacy has not kept pace with the expanding population of older adults [18,19,20]. A recent survey of American schools and colleges of pharmacy found that respondent schools spent a median of six contact hours teaching geriatric pharmacotherapy [21]. As a concept, deprescribing first appeared in the American College of Clinical Pharmacy (ACCP) Pharmacotherapy Didactic Toolkit in the recently published 2019 update as a Tier 1 topic (highest priority) [22,23]. Recently, clinical practice guidelines have developed processes to address the deprescribing of specific agents or classes of agents, however, there is no present guidance applying these guidelines to PharmD programs [17,24,25,26,27]. Zimmerman et al. conducted a survey including final-year trainees in medicine (n = 28), pharmacy (n = 35), and nursing (n = 11) to assess trainees’ preparedness, attitudes, and confidence toward deprescribing and perception of interprofessional roles in the process [28]. These authors found that pharmacy trainees perceived greater barriers to deprescribing than medicine trainees, and that both pharmacy and nursing students felt that their school’s curricula prepared them to identify and deprescribe PIMs compared to medical students [28].

The added value that pharmacists can bring to the care of older adults underscores the importance of pharmacists entering clinical practice possessing the necessary skills to address issues related to polypharmacy and deprescribing [17]. This study first examined if students received instruction on deprescribing and how they were taught this topic (e.g., lecture, case problems, experiential). Next, students’ perceptions of their school’s curriculum on deprescribing, patient willingness to deprescribe, and barriers to deprescribing were examined, along with their attitudes, ability and confidence in deprescribing. Finally, students who had received deprescribing in their curriculum were compared to those that had not on perceptions of their school’s curriculum on deprescribing, patient willingness to deprescribe, and barriers to deprescribing, along with their attitudes, ability and confidence in deprescribing; this research is important because it contributes to the literature by describing if and how deprescribing is taught to pharmacy students.

## 2. Materials and Methods

### 2.1. Study Design and Participants

This was a cross-sectional study of PharmD students enrolled in their last two years of an Accreditation Council for Pharmacy Education (ACPE) accredited pharmacy program. Participants were recruited for this study from all ACPE-accredited pharmacy programs in the US as of April 2019 (n = 132). An email explaining the study and a link to the online survey instrument was sent to one faculty member specializing in geriatrics at each school with the request that the email and link be forwarded to the appropriate student email lists. Faculty members specializing in geriatrics were first identified through a review of contact information from the American Association of Colleges of Pharmacy’s (AACP) Geriatrics Special Interest Group. If no faculty member was identified, a manual search of the pharmacy program’s website was performed. If no such faculty member was found, the survey link was sent to the chair of the pharmacy practice department. Faculty members were asked to forward the email to students who were either completing their didactic training or their Advanced Pharmacy Practice Experiences (APPE) for the academic year of 2018–2019; for most programs, this includes students in the third (P3) and fourth (P4) professional years of the PharmD program. Exclusion criteria included non-accredited schools of pharmacy and incomplete survey responses. The survey remained open for four weeks between 13 June 2019 and 11 July 2019. A follow-up email was sent at three weeks to the faculty member if no responses were received from that institution. The survey instrument was administered via SurveyMonkey (SurveyMonkey Inc., San Mateo, CA, USA). The Institutional Review Board at the University at Buffalo approved this study in May 2019 (IRB#00004245).

### 2.2. Survey Development

To develop the survey instrument, a literature search was conducted in PubMed from inception to April of 2019 using a combination of the terms “deprescribing,” “education,” to identify how previous studies had assessed the constructs of attitudes, perceived ability, and confidence regarding deprescribing among healthcare providers and trainees [28,29]. A 38-item survey instrument was developed by the study investigators. Two investigators with expertise in geriatric pharmacy education drafted the survey questions through an iterative process and the final instrument was pilot tested with two student investigators prior to dissemination. The survey instrument is included in Appendix A.

The survey included 11 questions on background characteristics, including age, gender, race/ethnicity, plans upon graduation, year in pharmacy school, prior pharmacy work and experiential rotation experience, if instruction on deprescribing was received in required non-experiential coursework and elective non-experiential coursework, and the type(s) of instruction (e.g., lecture, case problems, experiential) (Appendix A.1). Respondent background information and curricular exposure to deprescribing were summarized with descriptive statistics.

The next 15 items used a five-point Likert scale (1 = strongly disagree, 2 = disagree, 3 = neither agree nor disagree, 4 = agree, 5 = strongly agree). The first three items were collected to characterize perceptions related to their school’s curriculum on deprescribing, patient willingness to deprescribe, and barriers to deprescribing (Appendix A.2.1). The mean and standard deviation are reported for each item. The following 12 items were grouped into three scales measuring student perceptions regarding deprescribing attitudes (n = 3) (Appendix A.2.2), ability (n = 5) (Appendix A.2.3), and confidence (n = 4) (Appendix A.2.4). A score for each scale was computed as the average of the responses that made up that scale [30]. A scale score was computed only for those respondents who had valid responses for at least 75% of the items in the scale. The final 12 items consisted of multiple-choice questions to assess students’ knowledge of polypharmacy and deprescribing. The first six items comprised the validated polypharmacy knowledge assessment instrument developed by Thomas et al. [31] (Appendix A.3.1). The final six items, multiple-choice questions, were developed by two study authors to further assess students’ knowledge regarding deprescribing (deprescribing knowledge assessment) (Appendix A.3.2).

### 2.3. Statistical Analysis

The reliability of each scale was estimated using Cronbach alpha (α). An alpha of 0.7 is considered minimally acceptable and over 0.80 is considered indicative of high internal consistency [32]. The means and standard deviations are reported for the scale and items. Cronbach α was estimated for each knowledge assessment individually, as well as across all 12 items. The scores for the knowledge assessments were reported as a percentage. The mean percent and standard deviation for each assessment was presented.

A variable was constructed to denote curricular exposure to deprescribing if students responded ‘yes’ to deprescribing being a part of their required non-experiential coursework or a part of the elective non-experiential coursework they participated in. Students that reported curricular exposure to deprescribing were compared to students with no exposure using a series of *t*-tests; a *p*-value less than 0.05 was considered statistically significant. The *t*-test comparisons were conducted on each of the three items that assessed perception of school’s curriculum on deprescribing, barriers to deprescribing and patient willingness to deprescribe, the deprescribing scales (attitude, ability, and confidence), and each of the knowledge assessments, along with a combined score using both knowledge assessments. Despite the ordinal nature of the Likert scales, the *t*-test was selected, as aggregated scale data have been shown to be able to be analyzed like continuous data [33,34,35]. Additionally, for the three items that were not analyzed as aggregated scales, previous studies have suggested that parametric tests may be appropriate when five or more categories are utilized as part of the scale [33,36]. Cohen *d* effect size, a measure of the number of standard deviations by which the scores differ, was calculated for each comparison. Cohen *d* values less than 0.2 were considered very small and meaningless; greater than 0.2 and up to 0.5 were considered small but meaningful; greater than 0.5 and up to 0.8 were considered medium; greater than 0.8 were considered large [37].

## 3. Results

### 3.1. Study Sample

Responses were received from 131 students from 13 schools/colleges of pharmacy. Forty responses were excluded for various reasons, including the student was not a P3 or P4 student (n = 16), a scale score could not be computed due to >75% of items from a scale missing a response (n = 12), and missing responses to knowledge assessment questions (n = 12). This resulted in a final sample of 91 students from 12 schools (one response was missing the institution). The number of responses per institution ranged from one to 38. Responses from three institutions made up 70% of the sample. The median (interquartile range; IQR) responses per institution was 3 (1,10). There were 66 third professional year students (72.5%) and 25 fourth professional year students (27.5%) in the sample. Table 1 summarizes the characteristics of the respondents. Respondents were mostly female (n = 64, 70.3%), with a majority of respondents having plans for residency and/or fellowship training after graduation (n = 55, 60.4%). The majority of respondents attended public institutions (n = 68, 75.6%), and the majority of survey respondents were from pharmacy schools located in the northeast (n = 56, 62.2%).

### 3.2. Deprescribing in School of Pharmacy Curricula

Of the 91 respondents, less than half reported instruction on deprescribing as part of their required non-experiential coursework (n = 42, 46.2%), and fewer (n = 32, 35.2%) reported instruction about deprescribing as part of the elective non-experiential coursework. Twenty students (22.0%) reported deprescribing in both required and elective coursework. Overall, there were 54 (59.3%) students that reported receiving curricular exposure (required or elective) and 37 (40.7%) that reported no curricular exposure; these groups were used in later comparisons. Of the students who responded that deprescribing was part of their coursework, the majority of respondents reported deprescribing instruction activities of lectures (68.1%) and patient-centered case problems (50.6%). Just over one-fifth of survey respondents (23.1%) reported that deprescribing was not included as any part of their pharmacy school’s education activities (Table 2).

### 3.3. Student Attitudes and Perceptions Regarding Deprescribing

**Figure 1:**
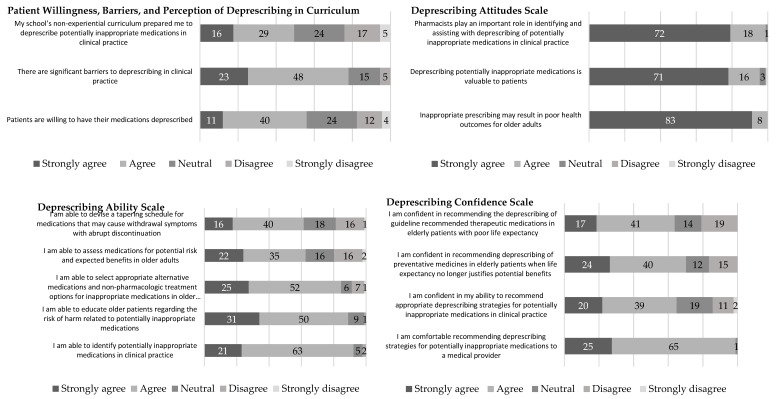
Frequency distribution for survey items on deprescribing.

The results for the perception of school’s curriculum on deprescribing, barriers to deprescribing, and patient willingness to deprescribe (Appendix A.2.1), along with the attitudes, ability, and confidence scales, are presented in Figure 1. The mean (out of 5) for the perception of school’s curriculum item, asking if students felt that their school’s non-experiential curriculum prepared them to deprescribe PIMs in clinical practice, was 3.4 (*SD* = 1.1), demonstrating generally low agreement with less than half (49.5%) of respondents selecting agree or strongly agree for this item. The mean of the item asking if there are significant barriers to deprescribing in clinical practice was 4.0 (*SD* = 0.8), indicating general agreement, with 78.1% of respondents selecting agree or strongly agree. The mean of the item asking if patients want their medications deprescribed was 3.5 (*SD* = 1.0), demonstrating generally low agreement, with approximately half (56.1%) of respondents selecting agree or strongly agree.

The deprescribing scales all had Cronbach alpha above 0.7, demonstrating acceptable consistency. The deprescribing attitude scale (Appendix A.2.2) had the highest scale mean (*M* = 4.8, *SD* = 0.4), indicating that, on average, respondents strongly agree with these items, while the deprescribing ability scale (Appendix A.2.3) mean (*M* = 3.9, *SD* = 0.6) was similar to the deprescribing confidence scale (Appendix A.2.4) mean (*M* = 3.8, *SD* = 0.7), indicating that, on average, respondents generally agree with these items. Overall, the items in the attitude scale had similar means and agreement levels (*M* ranged from 4.7 to 4.9). The items in the ability scale had more variation in means and agreement levels, with most students agreeing that they were able to identify inappropriate medications, educate older patients on risk of potentially inappropriate medications, and select appropriate alternatives (*M* ranged from 4.0 to 4.2), but were not as likely to endorse agreement that they were able to assess medications for potential risk and benefits or devise a tapering schedule for medications that may cause withdrawal symptoms (*M* was 3.6 for both items). On the confidence scale, there was variation in the means and agreement levels, with nearly all students agreeing or strongly agreeing that they were comfortable recommending deprescribing strategies for potentially inappropriate medications to a medical provider (*M* = 4.3, *SD* = 0.5), but respondents were not as confident in recommending deprescribing for elderly patients when life expectancy no longer justifies potential benefits (*M* = 3.8, *SD* = 1.0) or recommending deprescribing in elderly patients with poor life expectancy (*M* = 3.6, *SD* = 1.0).

### 3.4. Comparison of Perceptions and Knowledge Assessment Score Based on Curricular Exposure to Deprescribing

Table 3 summarizes the comparisons between students that received deprescribing curriculum exposure and those that did not. There were significant differences between the groups in both perception of deprescribing in the curriculum (*t*(89) = −2.26, *p* = 0.03) and patient willingness to deprescribe (*t*(89) = −2.39, *p* = 0.03); those with exposure to deprescribing had higher mean scores on both, which demonstrated a small but meaningful effect size (*d* = 0.48 and *d* = 0.49). Three scales were used to assess deprescribing attitudes, ability, and confidence, with the *t*-test, assessing differences between the two groups on the scales, which were not statistically significant (ability: *t*(89) = −0.46, *p* = 0.65; confidence: *t*(89) = −0.53, *p* = 0.60; attitudes: *t*(89) = −2.01, *p* = 0.07); however, it is noteworthy that the attitudes scale approached statistical significance and had a small but meaningful effect size (*d* = 0.41).

The mean percentage score on the six-item polypharmacy knowledge assessment instrument for the group with deprescribing curriculum exposure was 63.0% (*SD* = 23.3) and, for the group with no deprescribing curriculum exposure, the score was 58.1% (*SD* = 16.5). The mean percentage score on the 6-item deprescribing knowledge assessment for the group with deprescribing curriculum exposure was 67.3% (*SD* = 21.0) and, for the group with no deprescribing curriculum exposure, it was 60.4% (*SD* = 22.0). Neither assessment had adequate internal consistency with α < 0.3 for both instruments. There were no statistically significant differences in scores between the two groups. It is noteworthy that the *SD* was fairly high for both groups on each instrument (*SD*s ranged from 16.5 to 23.3) suggesting there is much variation around both instruments, which would impact both the α and *t*-test results. 

Combining the polypharmacy knowledge assessment instrument and the deprescribing knowledge assessment increased the α to 0.36, which is still low. The SD decreased for both groups for the combined assessment (*SD*s ranged from 16.2 to 16.4). The mean percentage score on the combined assessment for the group with deprescribing curriculum exposure was higher (*M* = 65.1%, *SD* = 16.2) than the group with no deprescribing curriculum exposure (*M* = 59.2%, *SD* = 16.4). The *t*-test results suggested that the difference approached statistical significance (*t*(89) = −1.69, *p* = 0.09) but did not meet the set threshold (*p* < 0.05), and the effect size (*d* = 0.36) was small but meaningful.

## 4. Discussion

### 4.1. Student Pharmacist Curricular Exposure to Deprescribing

Pharmacists play a key role in the practice of deprescribing and must be practice-ready in order to address the complex needs of an aging population. It has been suggested that a key change needed to fulfill this role is instruction on deprescribing in pharmacy education [16]. This exploratory study assessed if students were receiving instruction on deprescribing and the format of the instruction. In addition, it examined differences between students with deprescribing curriculum exposure and those with no exposure on their perceptions of deprescribing (in their curriculum, barriers, and patient willingness), on their deprescribing attitudes, ability and confidence, and on their knowledge of polypharmacy and deprescribing. The results contribute to the discussion regarding deprescribing education and can be used when developing the curriculum and activities on the topic.

In our study, less than half of the respondents (46.2%) reported that deprescribing was part of their required non-experiential coursework; some students reported participating in elective coursework on the topic (35.2%), yet many students reported no curricular exposure to deprescribing (40.7%). Respondents were asked which curricular experiences included deprescribing instruction; most selected lectures (68.1%), while a few selected Introductory Pharmacy Practice Experience (IPPE) (22.0%) or APPE (25.3%). Respondents were asked if their school’s non-experiential curriculum prepared them to deprescribe in clinical practice; this was more likely to be endorsed by students that received deprescribing curriculum exposure. The low percentage of students that reported deprescribing as part of their pharmacy school curriculum and the high percentage of content being delivered in lectures highlights the opportunity for growth and expansion of deprescribing education in required pharmacy school curricula [22,23].

### 4.2. Student Perceptions of Polypharmacy and Deprescribing

This study next examined students’ perceptions of deprescribing barriers and patient willingness, along with deprescribing attitudes, ability, and confidence; each of these were compared for students with deprescribing curriculum exposure and those with no exposure. Previous studies used the Patient Attitudes Toward Deprescribing, a validated questionnaire developed to assess patients’ willingness for and barriers to deprescribing, and found that between 51 and 92% of patients are willing to have medications deprescribed [38,39,40,41,42,43]. As pharmacy schools develop deprescribing principles into their curricula, interprofessional education and principles of shared decision making are imperative aspects to ensure that pharmacists are able to work with healthcare providers and patients to successfully deprescribe.

Similar to the Zimmerman et al. study, the pharmacy students in our study were likely to agree that there were significant barriers to deprescribing in clinical practice (78.1% agree or strongly agree) with no differences based on deprescribing curriculum exposure. In our study, just over half of all respondents (56.1%) agreed that patients would be willing to have their medications deprescribed, yet students with deprescribing curriculum exposure were more likely to agree than those with no deprescribing curriculum exposure. The *p*-value for the comparison of students with deprescribing curriculum exposure to those with no exposure on the deprescribing attitudes approached significance (*t*(89) = −2.01, *p* = 0.07), suggesting some differences (items included inappropriate prescribing may result in poor health, deprescribing is valuable to patients, and pharmacists play an important role in deprescribing), while there was no difference between the groups on the deprescribing ability and confidence scales.

The study by Zimmerman et al. described six major themes that healthcare students felt were necessary for successful deprescribing: curricular education and training, experiential education and training, knowledge and skills, interprofessional education, enhanced communication between providers, and resources for deprescribing [28]. As pharmacy programs look to enhance their deprescribing curricula, providing students with access to the previously listed resources and focusing on these themes may help to better prepare students to deprescribe in clinical practice. The results in our study suggest that students who received a deprescribing curriculum feel that patients are more willing to deprescribe and may also have a more favorable attitude toward deprescribing; however, their ability and confidence is similar to students who have not received deprescribing curricula. When viewing our results along with the six major themes above, it seems that most of the students in the study received instruction via lecture and case-based problems while few received experiential education and training (IPPE or APPE). The instruction may have impacted the students’ attitudes and view of patient perspective, yet the lack of clinical experience may limit their agreement with the ability and confidence scale items.

Nearly two-thirds of respondents felt confident in their ability to recommend appropriate deprescribing strategies for PIMs in clinical practice. In comparison, a survey given to physicians in Italy (n = 160), reported that 72% of physicians felt general confidence in their abilities to deprescribe [29]. A potential barrier to successful deprescribing identified in this prescriber survey was a lower percentage of respondents that were comfortable recommending the deprescription of medications complying to clinical practice guidelines at 53%. Just over 60% of respondents in our study agreed that they would be comfortable deprescribing guideline-directed therapy in patients with limited life expectancy. A similar percentage responded that they would be comfortable deprescribing guideline-directed therapy in patients with limited life expectancy. Studies have shown that guideline-recommended medications can safely be discontinued in those with limited life expectancy [44,45]. These results suggest that improving students’ ability to perform risk-benefit assessments of older adults’ medications may improve comfort with deprescribing.

### 4.3. Student Knowledge of Polypharmacy and Deprescribing

Thomas et al. developed the polypharmacy knowledge assessment instrument as a method of assessing baseline polypharmacy knowledge of healthcare providers and trainees [31]. The validation of the instrument was conducted with 74 healthcare professionals, including 37 internal medicine residents, 6 nurse practitioner (NP) residents, 9 primary care attending physicians, 12 pharmacists and pharmacy residents, and 10 geriatrics attending physicians and fellows. The average score on the instrument in that study was 92% for geriatricians, 86% for pharmacists, 59% for primary care attending physicians, and 54% for NP and MD residents. Cronbach alpha for the six-item version was 0.62. The instrument demonstrated the ability to distinguish between experts (geriatricians and pharmacists) and non-experts (primary care attendings, NP and MD residents). In our study, the polypharmacy knowledge assessment instrument, along with the study-developed deprescribing knowledge assessment, were used to assess student knowledge in this area. Both assessments had Cronbach alpha estimates below that considered acceptable. Since Cronbach alpha is a function of both covariances among items and the number of items in the analysis; increasing the number of items on the assessment may improve the Cronbach alpha, therefore, the assessments were combined but the Cronbach alpha was still below that considered acceptable. The polypharmacy knowledge assessment instrument was validated with a local sample that included health care professionals and trainees, which may have allowed for more knowledge on the topic and also less variation in knowledge. In our sample, it was apparent, especially within the group of students with deprescribing curriculum exposure, that there was a fair amount of variation (*SD* = 23.3); this suggests that the knowledge on deprescribing among students exposed to the topic is disparate, which may be a result of variation in the way it is taught in the curriculum. It is also important to note that when the two assessments are combined, the Cronbach alpha does increase, the SD decreases, and the difference on this knowledge assessment approaches statistical significance. This finding suggests that it will be necessary to develop better teaching methods and assessment tools related to deprescribing for all professionals throughout the continuum of medical education in order to advance the field [27].

### 4.4. Limitations and Future Directions

There are several limitations of the study that must be mentioned. The survey was sent to all ACPE accredited pharmacy programs, but usable responses were only received from a small percentage. As such, these results are not generalizable to all schools of pharmacy but should serve to prompt schools to assess how deprescribing is taught in their curriculum. Deprescribing is a complicated concept and students’ knowledge of deprescribing was assessed using the polypharmacy knowledge assessment instrument and deprescribing knowledge assessment. Minimizing the amount of knowledge-based content on the survey was done to encourage a higher response rate, but this approach may be limited in its ability to assess a student’s knowledge of deprescribing. Additionally, while the polypharmacy knowledge assessment instrument had acceptable internal consistency and known groups’ validity among healthcare practitioners in a previous study, this was not the case for the students in this study. Further evaluation of assessments of deprescribing knowledge are needed for student pharmacists. Finally, this study relied on students to report on curricular experiences and knowledge and these may not be entirely accurate. Future studies are needed to determine if the addition of specific deprescribing objectives and outcome measures for didactic and experiential training is warranted.

## 5. Conclusions

A slight majority of PharmD students reported deprescribing exposure in their curriculum and the majority of these reported instruction through lectures. Less than half of students felt that their didactic training adequately prepared them for deprescribing in the clinical setting. Student’s attitudes toward deprescribing were generally positive, but there was more variability in their perceptions of their ability and confidence in deprescribing. Students who were exposed were more likely to agree that their school’s curriculum prepared them to deprescribe and that patients were willing to deprescribe. This study reveals areas for improvement in the incorporation of deprescribing into professional pharmacy curricula.

## Figures and Tables

**Table 1:** Baseline characteristics of student respondents.

Characteristic	n (%)
**Professional Year**	
P3	66 (72.5)
P4	25 (27.5)
**Institution Type ^a^**	
Public	68 (75.6)
Private	22 (24.4)
**Region**	
South	15 (16.7)
West	8 (0.1)
Northeast	56 (62.2)
Midwest	11 (12.2)
**Gender**	
Male	27 (29.7)
Female	64 (70.3)
**Race/Ethnicity**	
White/Caucasian	67 (73.6)
Black or African American	1 (1.1)
Hispanic, Latino, or Spanish origin	2 (2.2)
Asian	16 (17.6)
Other	5 (5.5)
**Plans for further education upon your graduation ^b^**	
Residency or fellowship program	55 (60.4)
Additional academic degree	7 (7.7)
No plans	34 (37.4)
**Plans for primary employment upon graduation ^b^**	
Chain/Independent community pharmacy	39 (42.9)
Hospital	47 (51.7)
Clinic-based Pharmacy	35 (38.5)
Academia	13 (14.3)
Pharmaceutical Industry/government agency	7 (7.7)
Managed Care/Consultant/Administration	15 (16.5)
Nursing home/long-term care/home Care	9 (9.9)
Other	6 (6.6)
**Previous pharmacy experience ^b^**	
Community pharmacy	91 (100.0)
Hospital	86 (94.5)
Clinic-based Pharmacy	45 (49.5)
Nursing home/hospice/home care	34 (37.4)
Academia	8 (8.8)
Managed Care	20 (22.0)

Abbreviations: P3, third professional year; P4, fourth professional year; IPPE, Introductory Pharmacy Practice Experience; APPE, Advanced Pharmacy Practice Experience. ^a^ Institutional information available for 90 of 91 respondents. ^b^ Students were able to select multiple responses. Responses for these sections were not mutually exclusive.

**Table 2:** Student reported exposure to deprescribing in their institution’s curriculum.

	n (%)
**Instruction on deprescribing**	
Required coursework ^a^	42 (46.2)
Elective coursework ^b^	32 (35.2)
Both required and elective	20 (22.0)
None	37 (40.7)
**If instruction on deprescribing was part of the curriculum (didactic or experiential) at your school/college of pharmacy, during what curricular activities did this occur? ^c^**	
Lectures	62 (68.1)
Patient-centered case problems	46 (50.6)
Clinical simulations	19 (20.9)
Community projects, service learning	7 (7.7)
Online coursework	5 (5.5)
Clinical labs	10 (11.0)
IPPE	20 (22.0)
APPE	23 (25.3)
Research or Capstone projects	4 (4.4)
Other	2 (2.2)
Did not cover deprescribing in any education activities	21 (23.1)

Abbreviations: IPPE, Introductory Pharmacy Practice Experience; APPE, Advanced Pharmacy Practice Experience. ^a^ Required courses are those necessary for students to complete in order to receive their degree. ^b^ Elective courses are optional offerings that students may complete during their training. ^c^ Students were able to select multiple responses. Responses for these sections were not mutually exclusive.

**Table 3:** Analysis of pharmacy student’s attitudes and knowledge assessment scores based of didactic exposure to deprescribing.

	Cronbach Alpha (α) ^a^	Deprescribing Curricular Exposure (n = 54) Mean (SD)	No. Deprescribing Curricular Exposure (n = 37) Mean (SD)	Total (n = 91) Mean (SD)	*p*-Value	Cohen’s d	Magnitude of Effect ^b^
Perception of Deprescribing in Curriculum	-	3.6 (1.1)	3.1 (1.1)	3.4 (1.1)	0.03 *	0.48	small
Barriers to Deprescribing	-	4.0 (0.8)	4.0 (0.7)	4.0 (0.7)	0.83	0.05	very small
Patient Willingness to Deprescribe	-	3.7 (0.8)	3.2 (1.2)	3.5 (1.0)	0.03 *	0.49	small
Deprescribing Attitudes Scale	0.70	4.9 (0.3)	4.7 (0.5)	4.8 (0.4)	0.07	0.41	small
Deprescribing Ability Scale	0.79	3.9 (0.6)	3.9 (0.6)	3.9 (0.6)	0.65	0.10	very small
Deprescribing Confidence Scale	0.82	3.9 (0.7)	3.8 (0.8)	3.8 (0.7)	0.60	0.11	very small
Polypharmacy Knowledge Assessment Instrument ^a^ (%)	0.19	63.0 (23.3)	58.1 (16.5)	61.0 (20.8)	0.25	0.24	small
Deprescribing Assessment ^b^ (%)	0.27	67.3 (21.0)	60.4 (22.0)	64.5 (21.5)	0.14	0.32	small
Combined Assessments ^c^ (%)	0.36	65.1 (16.2)	59.2 (16.4)	62.7 (16.4)	0.09	0.36	very small

^a^ Cronbach alpha (a) reliabilities of 0.70 or above are considered generally acceptable for evidence of reliability for instruments with low stakes. ^b^ *d* < 0.2 is considered a very small effect size; *d* between 0.2 and 0.5 is considered small, *d* between 0.5 and 0.8 is considered medium, and *d* > 0.8 is considered large. Ref.: Thomas et al. *American Journal of Pharmaceutical Education*. 2017;83(5): 6435. * *p* < 0.05.

## Appendix A. Survey Instrument

### Appendix A.1. Demographics and Curricular Exposure to Deprescribing

1. At what school or college of pharmacy are you enrolled?
2. How do you describe yourself?

	Male
	Female
	Prefer not to list
	Other:

3. What is your race/ethnicity? (select all that apply)

	American Indian or Alaska Native
	Native Hawaiian or other Pacific Islander
	Asian
	White/Caucasian
	Black or African American
	Hispanic, Latino, or Spanish origin
	Other, please specify:

4. What are your current plans upon your graduation from your college/school of pharmacy (select all that apply)

	Pharmacy Residency Program
	Pharmacy Ph.D Program
	JD or Other Law
	Masters; please specify
	Other Health Professions (MD, DDS, DVM, etc.)
	Non -pharmacy Ph.D Program
	Fellowship
	No Plans for further education in the coming year.

5. What are your current plans for primary employment upon your graduation from your college/school of pharmacy? (check all that apply)

	Chain/Independent community pharmacy
	Hospital
	Clinic-based pharmacy
	Academia
	Pharmaceutical Industry/government agency
	Managed care/consultant/administration
	Nursing home/Long term care facility/Home care
	Other Pharmacy Related Field; please specify (Allow for free text)

6. Please select the statement that best describes your stage of training at your school/college of pharmacy.

	I am finishing the didactic portion of the curriculum and starting Advanced Pharmacy Practice Experiences (APPE) in the 2019-2020 academic year
	I am currently completing my Advanced Pharmacy Practice Experiences (APPE)
	I am currently completing only didactic courses and/or Introductory Pharmacy Practice Experiences (IPPE)
	Other, Please specify (Allow for free text)

7. Please select from the following settings in which you have had paid pharmacy work experience of greater than 1 month, completed Introductory Pharmacy Practice Experience (IPPE) rotations, or Advanced Pharmacy Practice Experience (APPE) rotations. (Select all that apply)

Previous Pharmacy work experience greater than one month	Previous Introductory Pharmacy Practice Experience (IPPE) rotations	Previous Advanced Pharmacy Practice Experience (APPE) rotations
□ Chain community pharmacy	□ Chain community pharmacy	□ Chain community pharmacy
□ Independent community pharmacy	□ Independent community pharmacy	□ Independent community pharmacy
□ Hospital	□ Hospital	□ Hospital
□ Clinic-based pharmacy	□ Clinic-based pharmacy	□ Clinic-based pharmacy
□ Hospice/Palliative care	□ Hospice/Palliative care	□ Hospice/Palliative care
□ Nursing home/Long term care facility	□ Nursing home/Long term care facility	□ Nursing home/Long term care facility
□ Home care	□ Home care	□ Home care
□ Academia	□ Academia	□ Academia
□ Managed care	□ Managed care	□ Managed care

8. Was there instruction on deprescribing as part of your REQUIRED pharmacy curriculum?

	Yes
	No
	Don’t know

9. Was there instruction on deprescribing as part of the elective coursework at your college/school of pharmacy?

	Yes
	No
	Don’t know

10. If instruction on deprescribing was part of the curriculum (didactic or experiential) at your school/college of pharmacy, during what curricular activities did this occur? (Select all that apply)

	Lectures
	Patient-centered case problems
	Clinical simulations
	Community projects, service learning
	Online coursework
	Clinical labs
	IPPE
	APPE
	Research or Capstone projects
	Other, please specify (Allow for free text)
	Did not cover deprescribing in any educational activities

On a scale from 5 = strongly agree to 1 = strongly disagree, please indicate your level of agreement with the following statements at the present time, assuming you had access to the patient, clinical assessments, and laboratory values:

### Appendix A.2.

#### Appendix A.2.1. Patient Willingness, Barriers, and Perception of Deprescribing in Curriculum

1. My school’s non-experiential curriculum prepared me to deprescribe potentially inappropriate medications in clinical practice
2. There are significant barriers to deprescribing in clinical practice
3. Patients are willing to have their medications deprescribed

#### Appendix A.2.2. Deprescribing Attitudes Scale

4. Inappropriate prescribing may result in poor health outcomes for older adults
5. Deprescribing potentially inappropriate medications is valuable to patients
6. Pharmacists play an important role in identifying and assisting with deprescribing of potentially inappropriate medications in clinical practice

#### Appendix A.2.3. Deprescribing Ability Scale

7. I am able to identify potentially inappropriate medications in clinical practice
8. I am able to educate older patients regarding the risk of harm related to potentially inappropriate medications
9. I am able to select appropriate alternative medications and non-pharmacologic treatment options for inappropriate medications in older adults
10. I am able to assess medications for potential risk and expected benefits in older adults
11. I am able to devise a tapering schedule for medications that may cause withdrawal symptoms with abrupt discontinuation

#### Appendix A.2.4. Deprescribing Confidence Scale

12. I am comfortable recommending deprescribing strategies for potentially inappropriate medications to a medical provider
13. I am confident in my ability to recommend appropriate deprescribing strategies for potentially inappropriate medications in clinical practice
14. I am confident in recommending deprescribing of preventative medicines in elderly patients when life expectancy no longer justifies potential benefits
15. I am confident in recommending the deprescribing of guideline recommended therapeutic medications in elderly patients with poor life expectancy

### Appendix A.3.

#### Appendix A.3.1. Polypharmacy Knowledge Assessment Instrument

1. Which of the following statements about sulfonylureas is true?

	Their effects diminish over time
	Glipizide has active metabolites and should be avoided in kidney disease
	They do not increase the risk of hypoglycemic events
	They decrease carbohydrate breakdown

2. A 75-year old man takes warfarin for atrial fibrillation and wishes to reconsider the risks and benefits of anticoagulation. Which of the following statements is true?

	The CHA2DS2-VASc and HAS BLED scores both estimate the risk of stroke
	Gait impairment resulting in a fall is a contraindication to warfarin use
	Bleeding events are generally more devastating than strokes
	Poor nutrition may increase the risk of adverse effects from warfarin

3. Which of the following is correct about age-related changes?

	The total body water increases and fat content decreases with age
	Decreases in lean muscle mass result in decreased creatinine production
	Medications are absorbed at the same rate, but to a lesser extent
	CYP 450 metabolism decreases predictably by 5% each year

4. Which of the following interactions correctly describes why a patient taking warfarin may have difficulty maintaining INR levels within therapeutic range?

	St. John’s wort decreases sensitivity to warfarin
	Ciprofloxacin decreases sensitivity to warfarin
	Green, leafy salads increase sensitivity to warfarin
	Vitamin K increases sensitivity to warfarin

5. A 70-year old woman has been taking omeprazole for years. She denies any heartburn in the past 6 months or any history of gastrointestinal bleeding. In your conversation with her, what concern related to adverse effects of proton pump inhibitors might you mention?

	Increased risk of clostridium difficile-associated diarrhea
	Increased risk of esophageal cancer
	Increased risk of osteonecrosis
	Increased risk of impaired glucose tolerance

6. A 70-year old woman takes temazepam every night for insomnia, but she heard about safety issues surrounding the drug and wants to discontinue it. She states she has tried sleep hygiene counseling in the past without much success. Anticipating that tapering off temazepam will be difficult for her, what would you recommend as adjunctive therapy during the taper?

	Prescribe lorazepam because it is safer than temazepam
	Advise her to repeat sleep hygiene counseling
	Refer her to a health psychologist for cognitive behavioral therapy
	Prescribe zolpidem because it is safer than temazepam

Reproduced with permission from Thomas et al., *American Journal of Pharmaceutical Education* published by the American Association of Colleges of Pharmacy, 2017.

#### Appendix A.3.2. Deprescribing Multiple Choice Questions

1. For an older patient who complains of chronic mild low back pain, which of the following medications would you NOT target for deprescribing from his regimen?

	Cyclobenzaprine
	Acetaminophen
	Celecoxib
	Ibuprofen

2. Zolpidem has been identified as a drug to avoid in the elderly. Which of the following is an acceptable alternative?

	Temazepam
	Diphenhydramine
	Quetiapine
	None of the above

3. Which of the following are considered potentially inappropriate for older patients due to anticholinergic activity?

	Citalopram
	Sertraline
	Paroxetine
	Trazodone

4. When recommending deprescribing of alprazolam 0.5mg four times daily in patient of 65 years of age, which of the following is the BEST initial approach?

	Reduce to alprazolam 0.5 mg three times daily
	Reduce to alprazolam 0.25 mg four times daily
	Convert to diazepam 2 mg three times daily
	Convert to lorazepam 0.5 mg four times daily

5. In a patient recently discharged home from the hospital where he was in the intensive care unit for 2 weeks, which of the following strategies should be used to deprescribe omeprazole 40mg daily which was started for stress ulcer prophylaxis?

	Discontinue omeprazole, start misoprostol 100 mcg four times daily
	Reduce omeprazole to 20 mg daily
	Discontinue omeprazole, start ranitidine 150 mg twice daily
	Discontinue omeprazole

6. Antipsychotics should not be used on older patients with dementia. Which of the following is the rationale behind this black box warning?

	Antipsychotics increase the risk of death
	Antipsychotics cause tardive dyskinesia
	Antipsychotics cause neuroleptic malignant syndrome
	Antipsychotics cause parkinsonian symptoms
